# microRNA Expression Profile of Purified Alveolar Epithelial Type II Cells

**DOI:** 10.3390/genes13081420

**Published:** 2022-08-10

**Authors:** Stefan Dehmel, Katharina J. Weiss, Natalia El-Merhie, Jens Callegari, Birte Konrad, Kathrin Mutze, Oliver Eickelberg, Melanie Königshoff, Susanne Krauss-Etschmann

**Affiliations:** 1 Institute for Lung Biology and Disease, Ludwig-Maximilians University Hospital Munich, Asklepios Clinic Gauting and Helmholtz Zentrum München, Comprehensive Pneumology Center Munich, Max-Lebsche-Platz 31, 81377 Munich, Germany; 2 Helmholtz Zentrum München, Department Strategy, Programs, Resources, Helmholtz Zentrum München German Research Center for Environmental Health, Ingolstädter Landstraße 1, 85764 Neuherberg, Germany; 3 Dr. von Hauner Children’s Hospital, Ludwig-Maximilians-University, 80337 Munich, Germany; 4 Early Life Origins of Chronic Lung Disease, Research Center Borstel, Leibniz Lung Center, Member of the German Center for Lung Research (DZL) and the Airway Research Center North (ARCN), 23845 Borstel, Germany; 5 Helmholtz Zentrum Munich, Lung Repair and Regeneration, Comprehensive Pneumology Center, Member of the German Center for Lung Research, 81377 Munich, Germany; 6 Evangelisches Krankenhaus Bergisch Gladbach, Ferrenbergstraße, 51465 Bergisch Gladbach, Germany; 7 Division of Pulmonary, Allergy and Critical Care Medicine, Department of Medicine, University of Pittsburgh Medical Center, Pittsburgh, PA 15261, USA; 8 Institute for Experimental Medicine, Christian-Albrechts-Universität zu Kiel, 24118 Kiel, Germany

**Keywords:** alveolar epithelial type II cells, type II pneumocytes, ATII, AECII, flow cytometry, autofluorescence, miRNAs, pathway analysis, TGF-beta, homeostasis, EMT

## Abstract

Alveolar type II (ATII) cells are essential for the maintenance of the alveolar homeostasis. However, knowledge of the expression of the miRNAs and miRNA-regulated networks which control homeostasis and coordinate diverse functions of murine ATII cells is limited. Therefore, we asked how miRNAs expressed in ATII cells might contribute to the regulation of signaling pathways. We purified “untouched by antibodies” ATII cells using a flow cytometric sorting method with a highly autofluorescent population of lung cells. TaqMan^®^ miRNA low-density arrays were performed on sorted cells and intersected with miRNA profiles of ATII cells isolated according to a previously published protocol. Of 293 miRNAs expressed in both ATII preparations, 111 showed equal abundances. The target mRNAs of bona fide ATII miRNAs were used for pathway enrichment analysis. This analysis identified nine signaling pathways with known functions in fibrosis and/or epithelial-to-mesenchymal transition (EMT). In particular, a subset of 19 miRNAs was found to target 21 components of the TGF-β signaling pathway. Three of these miRNAs (miR-16-5p, -17-5p and -30c-5p) were down-modulated by TGF-β1 stimulation in human A549 cells, and concomitant up-regulation of associated mRNA targets (BMPR2, JUN, RUNX2) was observed. These results suggest an important role for miRNAs in maintaining the homeostasis of the TGF-β signaling pathway in ATII cells under physiological conditions.

## 1. Introduction

MicroRNAs (miRNAs) are small, endogenous non-coding RNA molecules that regulate the expression of ~60% of the human genome by post-transcriptional inhibition of target mRNAs [1,2,3]. Since their discovery in 1993, interest in miRNAs has increased, uncovering their importance in physiological processes, such as metabolism, growth, cell signaling, inflammation and cell differentiation, as well as their implication in the pathogenesis of several diseases [4,5,6,7].

It has been shown that miRNAs play an important role in lung development and in the maintenance of pulmonary homeostasis, which is vital for the preservation of normal lung function and health [8,9]. Moreover, miRNAs could regulate the interplay of epithelial cells with other cell types through targeting of multiple pulmonary pathways [10,11,12,13,14]. Taking into consideration that miRNAs affect the expression of a large part of the genome, the detrimental dysregulation of miRNAs disrupts lung homeostasis and initiates disease pathogenesis. Indeed, there is emerging evidence that altered miRNA expression in respiratory diseases modulates disease phenotypes and ultimately disease progression. For example, members of the miR-200 and miR-29 families are down-regulated in models of idiopathic pulmonary fibrosis (IPF) [15,16], and miR-154 has been suggested to promote fibrosis in interstitial lung disease [17]. Furthermore, the dysregulation of miRNAs was associated with an increased asthma exacerbation risk [18], as well as with the pathogenesis of COPD [19,20]. Therefore, expression of miRNAs maintains tissue homeostasis, whereas when miRNA expression is dysregulated, pathological changes occur. It has been suggested that some alveolar epithelial type II (ATII) cell-derived miRNAs could play a role in the maintenance of alveolar homeostasis in response to injury [21].

Two types of epithelial cells—alveolar epithelial type I (ATI) and type II (ATII) cells—form the alveolus. ATI cells cover ~95% of the alveolar surface to mediate gas exchange and maintain barrier integrity [22,23]. In lung injury, dying ATI cells slough off, leading to increased permeability [23,24,25]. ATII cells, in turn, orchestrate re-epithelialization and function as progenitors for dead ATI cells, thus restoring barrier function and gas exchange [26,27,28,29,30,31]. In mature lungs, proliferation and turnover of cells is relatively low, with an estimate of 28–35 days for ATII cells. However, this kinetics is enhanced in response to lung injury [32,33,34]. On the other hand, failure to repair injured alveolar epithelium is associated with progression and initiation of many pulmonary diseases [35,36]. Studies reported that epithelial destruction and ATII cell apoptosis are critical hallmarks in many pulmonary diseases [37,38,39,40]. ATII cells, which cover only ~5% of the alveolar surface [41], maintain the homeostasis of the alveolus [42,43,44,45]. This later role is carried out by surfactant proteins SP-A, SP-B, SP-C, and SP-D, which are secreted by lamellar bodies within ATII cells, the only lung epithelial cells which produce and secrete all four surfactant proteins [46,47]. Moreover, surfactant proteins A and D play an additional role in host defense and regulation of immune responses [48,49,50]. Therefore, any deficiencies or mutations in surfactant protein synthesis result in the disruption of lung homeostasis [51,52,53,54,55,56]. Taken together, it is clear that ATII cells exert several important biological functions and thus are critical for the maintenance of alveolar homeostasis and promoting pulmonary health [32,57,58]. However, this contrasts with the limited knowledge of the expression of the miRNAs and miRNA-regulated networks which control homeostasis and coordinate diverse functions of murine ATII cells.

The goal of this study was, therefore, to identify a set of miRNAs that are critical for maintenance of ATII cell homeostasis. These miRNAs could be further used to design miRNA-based therapeutics that target their function. The goal of this study was to identify miRNAs expressed by murine ATII cells under normal, non-pathologic conditions and to elucidate potential miRNA-controlled pathways of ATII cell homeostasis. We assumed that every method for isolating ATII cells will bias at least some microRNAs to some extent. To circumvent this problem, we decided to use ATII cells obtained by two different isolation procedures (panning and sorting) and to use the cut set of expressed microRNAs expressed by both sATII and pATII. We aimed to identify miRNAs that are expressed in all kinds of putative ATII cell subsets and not in a subset that might be enriched by a single method. To this end, we used two different methods (panning and sorting) for the purification of ATII cells to avoid bias in the miRNA composition introduced by a single method. For this purpose, a three-step approach was followed: first, a protocol for the isolation of highly pure murine ATII cells was developed using fluorescence-activated cell sorting (FACS) for further miRNA profiling. Second, we intersected miRNA profiles from our FACS-based procedure with those obtained by a previously published protocol relying on negative selection of ATII cells in antibody-coated plastic dishes [57]. Third, we used this dataset for in silico pathway enrichment analysis of ATII miRNA targets. In silico target prediction tools for miRNAs are highly prone to false-positive results. We therefore restricted the pathway analyses to miRNA–target pairs that have been confirmed previously and are available through the Ingenuity^®^ software. Finally, we corroborated our findings in human epithelial alveolar cell line A549. The study limitations include the fact that impurities in the panned cell fraction were mostly due to contamination with CD31- and CD45-postive cells, while the sorted cells had a very low number of contaminating cells of unknown composition, which might have been due to ATII progenitor cells.

## 2. Materials and Methods

### 2.1. Animals

Female C57BL/6NCrl wild type 6–12-week-old mice (5 mice per group) were maintained under specific pathogen-free conditions in individually ventilated cages. Mice were fed fortified rodent chow and water *ad libitum*. All animal experiments were approved by the Animal Ethics Committee of the government of Upper Bavaria, and all animal studies were conducted in compliance with the guidelines of the Institutional Animal Care and Use Committee of the Helmholtz Center Munich, Bavaria, Germany.

### 2.2. Preparation of Single-Cell Suspensions and Cell Sorting

Single-cell suspensions were prepared as described previously [58] from the whole lungs of female C57BL/6 mice (6–12 w). The mice were anesthetized by intraperitoneal injection of MMF (Medetomidine 0.5 µg/g, Midazolam 5.0 µg/g, Fentanyl 0.05 µg/g) and 60 µL of heparin (for blood coagulation inhibition (5 IU/µL, Ratiopharm, Ulm, Germany)). Lungs were perfused via the right ventricle with 10 mL PBS and 1.5 mL Dispase (BD, CA) instilled over a tracheal catheter. This was followed by a 0.3 mL instillation of pre-warmed to 42 °C low-melt agarose (1%) (Invitrogen, Darmstadt, Germany). Lungs were removed and incubated for 45 min in 2.5 mL Dispase at room temperature. Then, lungs were transferred to a culture dish containing 5 mL medium (DMEM/F12 (1:1) (Gibco, Darmstadt, Germany) supplemented with 0.04 mg/mL DNase I (AppliChem, Darmstadt, Germany), 3.6 mg/mL D-(+)-Glucose (AppliChem, Germany) and 1% Penicillin/Streptomycin (PAA, Cölbe, Austria)), and divided into separate lobes. The lobes were then sequentially transferred into a new culture dish containing 8 ml of medium, where the tissue was gently teased apart with forceps. The resulting cell suspension was homogenized and transferred into a 50 mL conical tube. The cell suspension was serially filtered through 100, 20 and 10 μm nylon meshes and then centrifuged at 200× *g* for 10 min at 15 °C. The supernatant was discarded, and the cell pellet was resuspended in medium (DMEM/F12 (1:1), (Gibco) containing 3.6 mg/mL D-(+)-Glucose (AppliChem), 1% Penicillin/Streptomycin (PAA) and 2% FBS Gold (PAA)).

Thereafter, single-cell lung suspensions from 3–4 mice were incubated on ice with rat anti-mouse CD45-APC (IgG2b, κ; BD Pharmingen) and rat anti-mouse CD31-APC (IgG2a, κ; BD Pharmingen). The antibodies are listed in Table 1. Cells were then washed and resuspended to a final concentration of 10 × 10^6^/mL in DMEM/F12 (1:1) (Gibco, Germany) containing 2% FBS Gold (PAA, Cölbe, Austria). After serial filtration through 100, 40 and 35 µm cell strainers (BD Biosciences, Heidelberg, Germany), cells were sorted on a FACS Aria II (BD Biosciences). Cell doublets were excluded according to FSC-H to FSC-A and FSC-W to FSC-A characteristics. ATII cells were identified as the CD45/CD31-negative and autofluorescence (FITC channel)-high population. Cells were sorted using an 85 µm nozzle tip at 45 psi sheath fluid pressure. Cells isolated by this procedure were designated as sATII. For RNA isolation, sorted cells were immediately pelleted and stored at −80 °C. Cell sorting was performed immediately after cell extraction.

### 2.3. Flow Cytometry and Immunofluorescence Staining for Purity Assessment

Cells were stained with antibodies for 20 minutes on ice and expression markers were analyzed with a BD LSR II flow cytometer (BD Biosciences). For immunofluorescence staining, 1 × 10^5^ cells in 200 μL/chamber were used, and for flow cytometry, 1 × 10^5^ in 50 μL antibody were used. For intracellular staining, cells were fixed and permeabilized with IntraPrep (Beckman Coulter, Krefeld, Germany) according to the manufacturer’s protocol. For immunofluorescence staining, cells were centrifuged for 5 min at 200× *g* (4 °C) on culture slides using a Rotina 420R centrifuge (Hettich, Tuttlingen, Germany) and dried overnight. Cytospins were fixed with acetone:methanol (1:1) (AppliChem), blocked with 5% BSA (Sigma-Aldrich, Schnelldorf, Germany) in PBS and stained with primary and secondary antibodies (Table 1) diluted in 0.1% BSA in PBS. Cells were then fixed with 4% PFA (Microcos, Germany) and mounted with ProLong^®^ Gold antifade reagent with DAPI (Invitrogen). The antibodies used in this study are listed in Table 1. For analysis of dead cells, Propidium iodide (Sigma-Aldrich) was added for 10 min at 4 °C prior to the analysis by flow cytometry.

### 2.4. Isolation of ATII Cells (Panning)

Lung single-cell suspensions were prepared and primary ATII cell isolation by panning (designated pATII) was performed as described by Königshoff et al. [57]. Briefly, culture dishes coated with CD45 and CD16/32 antibodies (Table 1) (15 μL of each antibody/10 mL DMEM per culture dish) were incubated overnight at 4 °C and thereafter washed with 5 mL DMEM twice. Then, 5 mL of single-cell suspension was added to the coated dishes and incubated for 35 min at 37 °C in order to remove lymphocytes and macrophages. To allow the adherence of fibroblasts, the unattached cells were collected, transferred to new uncoated dishes and incubated for 35 min at 37 °C. The supernatant was then pooled and centrifuged at 15 °C for 10 min at 200 g. The pellet was stored at −80 °C for further RNA isolation and primary ATII cells were resuspended and processed for flow cytometry.

### 2.5. Papanicolaou Staining

PAP staining was performed as described by Dobbs LG [59]. In brief, cells were centrifuged on coverslips and dried overnight. The following day, cells were stained with hematoxylin and thereafter dipped in lithium carbonate solution. After incubation with increasing concentrations of ethanol, cells were immersed in xylene:ethanol 1:1 and thereafter rinsed with xylene. Afterwards, cells were embedded in Entellan.

### 2.6. TGF-β1 Stimulation of A549 Cells

Human alveolar epithelial A549 cells (ATCC, CCL-185™) were cultured in DMEM/F12 (Gibco) medium supplemented with 10% FBS Gold (PAA)_._ The cells were seeded into 6-well plates at a density of 2 × 10^5^ cells/well and incubated overnight at 37 °C in a humidified atmosphere at 5% CO_2_. Prior to the treatment, the cells were starved for 24 h in DMEM/F12 media containing 0.1% FBS and then treated for 72 h with either vehicle control (0.1% BSA in 4 mM HCl) or recombinant human TGF-β1 (2 ng/mL) (R&D Systems, USA). Thereafter, cells were lysed with Qiazol (Qiagen, Hilden, Germany) and the lysates were stored at −20 °C until RNA isolation. All stimulations were carried out in triplicate and repeated independently three times.

### 2.7. RNA Isolation

Total RNA, including miRNAs, was isolated from primary ATII and A549 cells using an miRNeasy miRNA purification kit (Qiagen) according to the manufacturer’s instructions. Total RNA concentration was quantified by absorbance at 26 0nm with a NanoDrop 1000 spectrophotometer (Thermo Scientific), and RNA integrity was assessed by agarose gel electrophoresis.

### 2.8. Reverse Transcription and Quantitative PCR of mRNAs

Reverse transcription was performed using random hexamers and MuLV reverse transcriptase according to the manufacturer’s instructions (Life Technologies, Darmstadt, Germany), with 350ng total RNA as input. Relative quantification of mRNA expression was performed using LightCycler^®^ 480 SYBR Green I Master Mix (Roche, Mannheim, Germany) with the LightCycler^®^ 480 II system (Roche). All primers had an amplification efficiency of ≥92.5% and Cq values were corrected for inter-run variations. The primer sequences are listed in Table 2. Cq values above 35 were regarded as not expressed. Transcript abundance was calculated using the ΔΔCq method [60]. For sATII cells, *Hprt* was used as a reference gene and *Sftpc* mRNA expression served as a calibrator. For A549 cells, the arithmetic mean of the Cq values for *HPRT1* and *RNA18S5* served as a normalizer. Outliers were excluded using a modified Z-score [61]. T-bars, representing the range of expression levels due to sample variation, were calculated as 2^−(ΔΔCq ± S). S (standard deviation of the ΔCq value) was calculated by the formula S = (s_1_^2 + s_2_^2)^1/2, where s_1_ and s_2_ are the SEMs of the Cq(*target*) and Cq(*reference*) values from four (sATII) or three (A549) independent experiments, with three technical replicates for each. Statistical significance was calculated using ΔCq values and unpaired *t*-tests (GraphPad Prism).

### 2.9. Reverse Transcription and Quantitative PCR of miRNAs

Quantification of miRNAs was performed using TaqMan^®^ miRNA assays (Life Technologies) and the TaqMan^®^ miRNA reverse transcription kit (Life Technologies), according to manufacturer’s instructions. MiR quantitation was performed on a LightCycler^®^ 480 II (Roche) instrument using TaqMan^®^ Universal Master Mix II, no UNG (Life Technologies). TaqMan^®^ miRNA assays used were: hsa-miR-16-5p (Assay ID 000391), hsa-miR-17-5p (Assay ID 002308), hsa-miR-24-3p (Assay ID 000402), hsa-miR-30c-5p, (Assay ID 000419) and RNU6B (Assay ID 001093). Relative transcript abundance levels and statistical significance were calculated as described for mRNAs, with the difference that RNU6B served as a reference gene and ΔCq values of vehicle control-treated A549 cells were used as a calibrator.

### 2.10. Analysis of TaqMan^®^ Real-Time PCR miRNA Array Data

Total RNA concentration was quantified using a NanoDrop 1000 instrument (Thermo Scientific), and RNA integrity was assessed with a Bioanalyzer 2100 instrument (Agilent, Stuttgart, Germany). Samples with OD260/280 ratio of ≥1.85 and with an RNA integrity number (RIN) of ≥6.5 were used for miR array studies. For miR cDNA synthesis, 135 ng total RNA was reverse transcribed using stem-loop MegaPlex RT primers (rodent pool sets A+B v3.0) and an miRNA reverse transcription kit on a PeqStar 96 thermal cycler (Peqlab, Erlangen, Germany). Pre-amplification of the RT product was performed using TaqMan^®^ PreAmp Master Mix and PreAmp Primer Mix (rodent pool sets A + B v3.0) on the 7900HT Fast RT-qPCR system. For miR expression profiling, TaqMan^®^ Array Rodent MiRNA A+B Card Sets v3.0 containing 641 TaqMan^®^ assays detecting mature murine miRNAs present in miRBase v15 were used65. Quantitative real-time PCR was performed on a 7900HT Fast RT-qPCR system using TaqMan^®^ Universal PCR Master Mix. Raw cycle threshold (Cq) values were determined using Sequence Detection Software (SDS) v2.4 and SDS RQ Manager 1.2.1 (Life Technologies, Germany) with automatic settings for baseline and threshold. MiRNA assays with replicate differences larger than one Cq were filtered out and miRNAs with Cq > 32 were regarded as not detectable and excluded from the analysis. Global mean normalization was used to determine normalized relative quantities (NRQs) 66. MiRNAs with |NRQ fold differences| of ≤1.5 (sorted vs. panned ATII cells) were regarded as similarly expressed in both cell preparations. Pathway enrichment analysis of ATII miR targets was carried out using the Ingenuity^®^ software. Target mRNAs were filtered using the Ingenuity^®^ miRNA target filter to contain only those mRNAs with previously confirmed miR seed–target interactions. The identified target mRNAs were then associated with the canonical pathway library contained in the Ingenuity^®^ Knowledge Base. The significance of the association between the dataset and a given canonical pathway was measured in two ways: (1) as a ratio of the number of molecules from the dataset that map to the pathway divided by the total number of molecules that map to the canonical pathway; and (2) Fisher’s exact test, with Benjamini–Hochberg (BH) correction for multiple testing, was used to calculate the *p*-value determining the probability that the association between the genes in the dataset could be explained by chance alone.

## 3. Results

### 3.1. Isolation by Sorting and Assessment of Purity of Primary Murine ATII Cells

**Figure 1:**
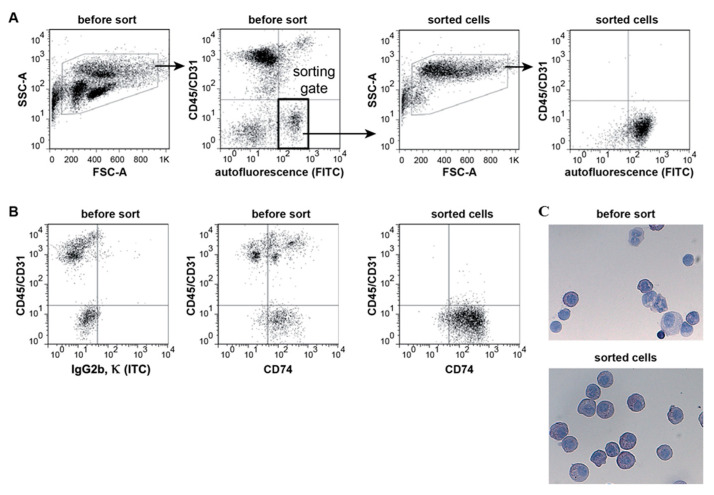
FACS strategy and purity of sorted ATII cells. (**A**) ATII cells were sorted based on high autofluorescence (FITC-channel) and absence of CD45 and CD31 surface expression (left panels). CD45 and CD31 expression levels were measured in the same channel (APC) and the gate used for sorting is highlighted as a thick rectangle. Sorted cells were re-analysed using the same gating strategy (right panels). Removal of doublets based on FSC characteristics not shown. Dot plots are representative of four independent experiments. (**B**) Representative dot plots of cells stained for CD45, CD31 and intracellular CD74 before and after sorting (ITC: isotype control). (**C**) Light microscopic images of Papanicolaou-stained cytospin preparations of cells before and after sorting (×400). ATII cells show characteristic dark blue inclusions in the cytoplasm (n = 4, mean ± SEM).

We developed a method for the isolation of highly purified “untouched by antibodies” primary ATII cells from murine lungs based on their autofluorescence [62,63] (Figure 1A). Critical steps included cell sorting of the ATII cell population based on its autofluorescence parameters measured in the FITC channel. ATII cells were isolated as negative for lineage markers of hematopoietic (CD45) and endothelial (CD31) cells (Figure 1A, left panels) defined as CD45/CD31-APC^negative^ and autofluorescence-FITC^high^. FSC served to remove doublet cells, while the APC channel was used as a dump channel for CD31^positive^ endothelial cells and CD45^positive^ leukocytes (mainly alveolar macrophages) using APC-conjugated antibodies for both of these antigens. Flow cytometric re-analysis showed that sorted cells remained highly autofluorescent (Figure 1A, right panels). Since ATII cells express MHC class II antigens and the associated invariant chain polypeptide CD74 [63,64,65], we investigated CD74 expression of the sorted cells as an indicator of ATII cell purity after the removal of CD45^positive^ cells, which are also known to express CD74 in the murine lung (Figure 1B).

Papanicolaou staining (Figure 1C) showed that nearly all of the sorted cells have dark blue inclusions in the cytoplasm (lamellar bodies)—a characteristic feature of ATII cells [59].

### 3.2. Confirmation of Epithelial and ATII Identity of the Sorted Cell Population

**Figure 2:**
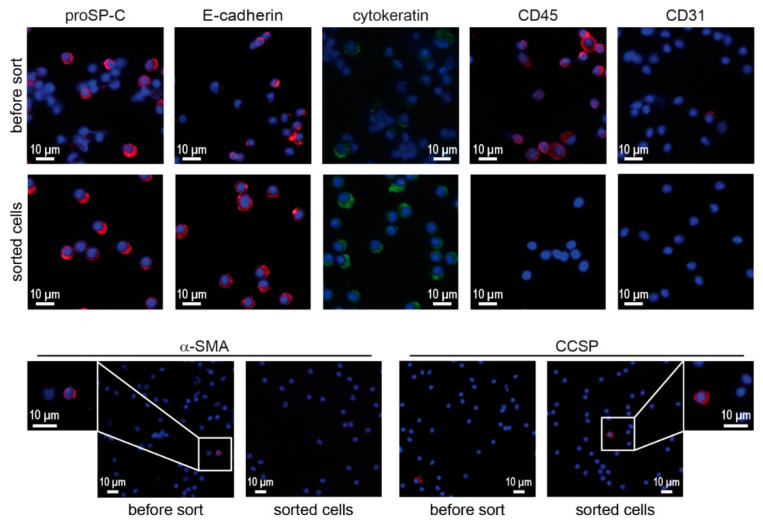
Immunofluorescence for phenotypic markers on cytocentrifuge preparations of lung cell suspensions (before sorting) and sorted cells. Cytocentrifuge preparations of whole lung cell suspensions and sorted cells were stained for phenotypic markers associated with ATII cells (proSP-C, E-cadherin, cytokeratin), leukocytes (CD45), endothelial cells (CD31), smooth muscle cells (α-SMA) and Club cells (CCSP). Scale bars represent 10 μm.

To further corroborate the identity of sorted ATII cells, cytocentrifuge preparations were stained with characteristic ATII and non-ATII phenotypic markers. Sorted cells were highly positive for pro-surfactant protein C (proSP-C) and epithelial cell marker proteins E-cadherin and cytokeratin (Figure 2). On the other hand, the expression of leukocyte marker CD45, endothelial marker CD31 and smooth muscle cell marker α-SMA was not detected in sorted cells. Some very few cells were found to express the Club cell secretory protein (CCSP) after sorting.

### 3.3. Viability, Purity and Phenotypes of ATII Cells Isolated by Sorting and Panning

Sorting and panning methods were compared based on cell viability (PI exclusion using flow cytometry) and purity (expression of phenotypic markers using flow cytometry and qRT-PCRs).

**Figure 3:**
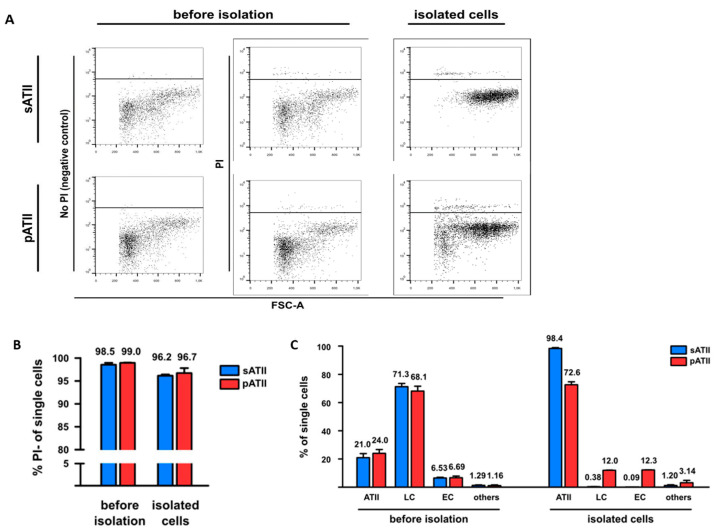
Viability, phenotypic markers and phenotypic expression of sATII and pATII cells before and after isolation. (**A**) Whole lung suspensions without PI staining were used as negative controls (left panels). Viable cells were identified for sATII (upper row) and pATII (lower row) in the whole lung suspension (before isolation) and isolated cells were identified by PI exclusion (middle and right panels). (**B**) Flow cytometric analysis of the viability of sATII and pATII cell populations before and after isolation as determined by propidium iodide (PI) negativity. (**C**) Purity of sATII and pATII cell preparation before and after isolation. ATII cells were defined as CD45/31^neg^ CD74^pos^ cells, leukocytes as CD45/31-APC^pos^ cells without CD31-PE^pos^ cells and endothelial cells as CD31-PE^pos.^ cells. Note that the CD31-PE antibody used recognizes a different epitope of CD31 than the CD31-APC antibody used for sorting. Each value is the mean of four independent experiments for sATII and two independent experiments for pATII. T-bars show the standard errors of the means (SEMs).

Viable cells were analyzed by flow cytometry as PI-negative. Propidium iodide (PI) exclusion (Figure 3A,B) confirmed the high viability of cell populations before isolation (PI: above 98%) and after isolation (PI: above 96%) for sATII and pATII, with a slightly higher viability (96.7%) for pATII.

To assess the purity (phenotypic markers) and to determine the fractions of non ATII cells, such as endothelial cells and leukocytes, sATII and pATII isolated cells were stained with a PE-conjugated antibody recognizing a different CD31 epitope [66] to the CD31-APC antibody used for sorting (Figure 3C). ATII cells were defined as CD45^neg^/CD31^neg^/CD74^pos^ cells, and their phenotype was confirmed by qRT-PCR (*Sftpc*). We observed that the percentage of ATII cells in sATII and pATII cell populations increased from 21.0% and 24.0% before the isolation to 98.4% and 72.6% in the isolated cells, respectively. However, the percentage of leukocytes (defined as CD45^pos.^ (APC) minus CD31^pos.^ (PE) cells) and endothelial cells (CD31^pos.^ (PE) cells) in the sATII population decreased from 71.3% and 6.53% before sorting to 0.38% and 0.09% after the cell sorting, respectively. By comparison, the percentage of leukocytes in the pATII cells decreased from 68.1% before isolation to 12.0% after isolation, while endothelial cells in pATII showed a relative increase from 6.69% before sorting to 12.3% in sorted cells (Figure 3B) in four independent experiments. Cells not expressing CD45, CD31 or CD74 were labeled as other.

### 3.4. MiRNA Expression Profiling of ATII Cells and Pathway Enrichment Analysis of Downstream mRNA Targets

**Figure 4:**
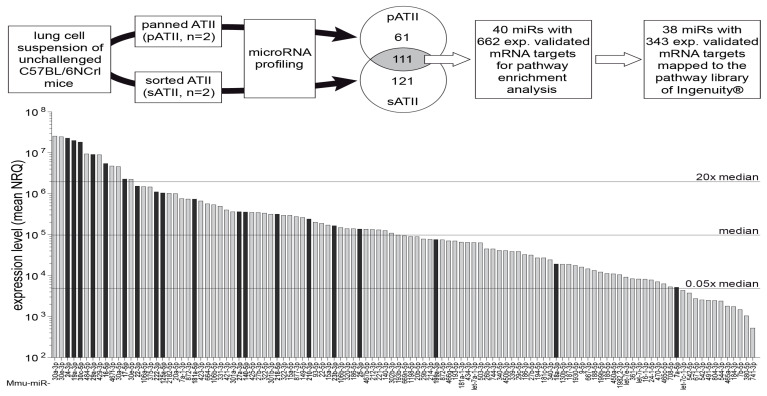
Workflow and miRNA expression profile of ATII cells. Lung single-cell suspensions were generated from unchallenged female C57BL/6NCrl mice. ATII cells were isolated either by negative selection (panning, pATII, n = 2) or by cell sorting (sATII, n = 2). Both cell preparations were subjected to miRNA profiling using TaqMan^®^ array microfluidic cards (Life Technologies). In pATII and sATII, respectively, 61 and 121 miRNAs had fold differences larger than 1.5 (FC > 1.5). A cut set of 111 miRNAs with similar expression levels (|fold difference| ≤1.5) in pATII and sATII was identified and is represented as a bar diagram. For 40 of these bona fide ATII miRNAs, experimentally observed interactions with 662 mRNA targets were available in the Ingenuity^®^ database and this information was used for pathway enrichment analysis, resulting in 38 miRNAs targeting 343 mRNAs in 145 pathways. Black bars indicate 19 miRNA targeting components of the TGF-β signaling pathway. The majority (16 out of 19) of these miRNAs were expressed above median level. A complete list of the miRNAs expressed in ATII cells is available as a spreadsheet file (see Table S2 supplementary material). n = 2 represents a biological replicate.

ATII cells obtained by sorting (sATII, n = 2 biological replicates) and panning (pATII, n = 2) were used for miRNA profiling, which showed an expression of 293 miRNAs at detectable levels. Of these, 111 miRNAs were expressed at similar levels (|FC| ≤ 1.5x) in both sATII and pATII preparations, and hence were termed ATII miRNAs (Figure 4) and further used for pathway enrichment analysis. To identify target mRNAs from these 111 miRNAs, we used Ingenuity^®^’s [67] miR–target filter restricted to experimentally observed miR–target interactions. By this means, we identified 40 ATII miRNAs with 662 previously validated mRNA interactions in the cut set of 111 ATII miRNAs. Of note, 38 of these miRNAs were associated with 343 mRNAs present in the canonical pathway library of Ingenuity^®^ (see Figure 4 for an overview of the workflow).

**Figure 5:**
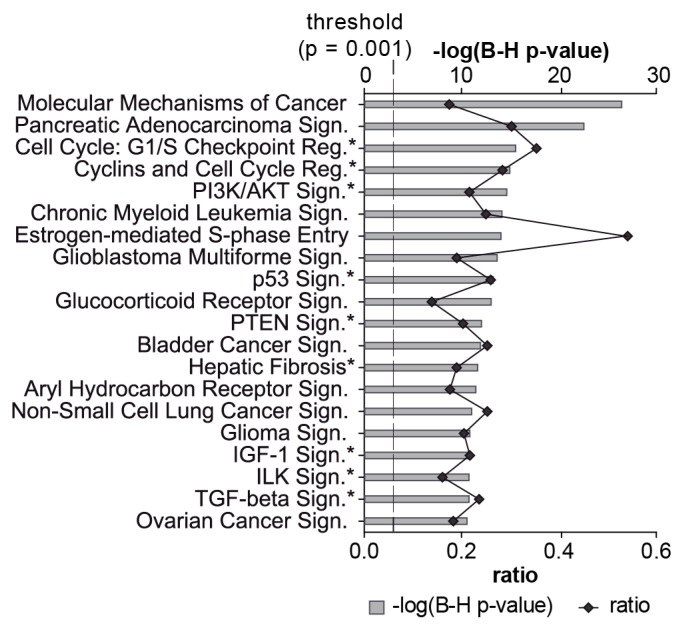
Top 20 enriched signaling pathways targeted by miRNAs expressed in ATII cells. The 111 miRNAs expressed at similar levels in pATII and sATII were used as inputs for the miRNA target filter module in Ingenuity^®^. The top 20 signaling pathways associated with the dataset are shown. The significance of this association is expressed by the probability (grey bars) that the association between the targets and the pathway is not due to chance (BH-adjusted *p*-value, Fisher’s exact test). The degree of miRNA interaction within a certain pathway was calculated as the ratio of the number of targets that map to a given pathway to the total number of molecules within the pathway (black line). The dashed line indicates the significance threshold at *p* = 0.001. An asterisk highlights signaling pathways that have been associated previously with fibrosis and/or EMT.

Significant enrichment (adj. *p*-value < 0.001) of 343 target mRNAs was observed in 143 signaling pathways and 2 metabolic pathways (nicotinate and nicotinamide metabolism, inositol phosphate metabolism). These pathways were assigned to 20 categories (Table 3). From the top 20 significant signaling pathways, 9 (marked with an asterisk*) have already been associated with fibrosis and/or EMT (e.g., PI3K/Akt, PTEN, IGF-1 and TGF-β) [68,69,70,71,72,73,74,75] (Figure 5). Another 9 of the top 20 pathways have been associated with cancer. Taken together, these results suggest an important role for ATII miRNAs in controlling cellular growth, proliferation and development.

### 3.5. Key Upstream Regulators of Target mRNAs

In the next step, we looked in silico for potential upstream regulators of all 662 target mRNAs, thereby identifying three miRNAs (16-5p, 30c-5p and 302d-3p) and two growth factors (TGFβ1 and EGF) as the top upstream regulators of our target mRNAs (see Table S1). Intriguingly, miR-16-5p and miR-30c-5p showed, also, very high expression levels (above 20× median) in the ATII expression profile (Figure 4, Table S2—Supplementary Material). TGF-β as well as EGF signaling pathways showed significant enrichment of ATII miRNA targets. Since deregulation of TGF-β signaling plays a crucial role in chronic lung diseases [76,77,78], we further focused on the investigation of ATII miRNAs in the TGF-β signaling pathway.

**Figure 6:**
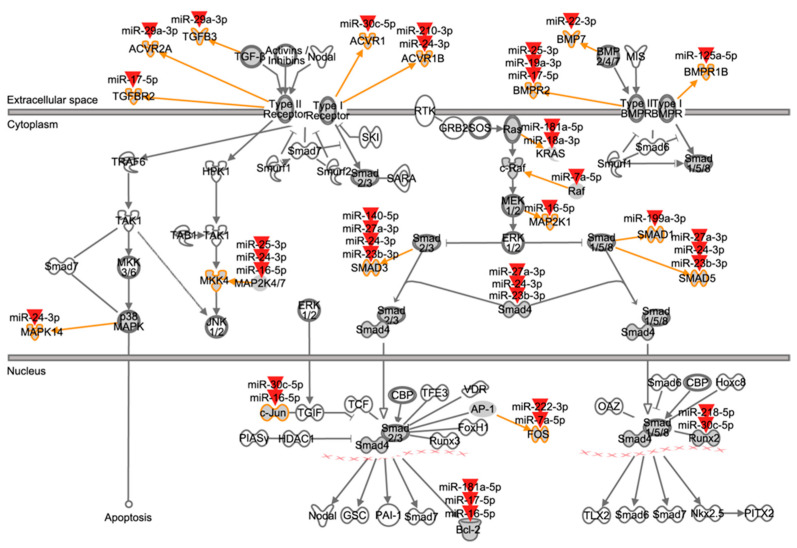
Mapping of ATII miRNAs to TGF-β signaling pathway components. This figure represents the canonical TGF-β signaling pathway from the Ingenuity^®^ pathway library. Orange arrows and outlines indicate ATII miRNA targeted members within molecule families (dark grey icons). Red arrowheads symbolize ATII miRNAs. For example, TGFBR2 is a member of the type II TGF-β receptors, and its mRNA is targeted by miR-17-5p. Compare Table 4 for an overview of interactions.

The canonical TGF-β signaling pathway of the Ingenuity^®^ pathway library consists of 89 molecules. Nineteen ATII miRNAs (16 of which were expressed above median level; see Figure 4 and Table S2—Supplementary Material) were found to target 21 TGF-β signaling components located at several levels in the pathway, from ligands to transcription factors and target genes (Figure 6 and Table 4). Eleven molecules within the TGF-β pathway were targeted by up to four miRNAs, and ten miRNAs targeted more than one TGF-β signaling molecule (Table 4). Overall, these findings indicate a tight regulation of this pathway in ATII cells by miRNAs and the fact that they are expressed under physiological conditions indicates a role for these miRNAs in the homeostasis of the TGF-β pathway.

### 3.6. mRNA Targets Located within the TGF-β Signaling Pathway

#### 3.6.1. Effect of TGF-β1 Treatment on miRNA Expression in A549 Cells

Our in silico analyses indicated that highly expressed ATII miRNAs inhibit TGF-β signaling. Hence, we hypothesized that TGF-β stimulation might induce a down-regulation of these inhibitory miRNAs and lead to de-repression of this pathway. Since primary ATII cells gradually lose their phenotypes in in vitro culture, we studied the effects of TGF-β stimulation on these inhibitory miRNAs in a human alveolar epithelial A549 cell line.

**Figure 7:**
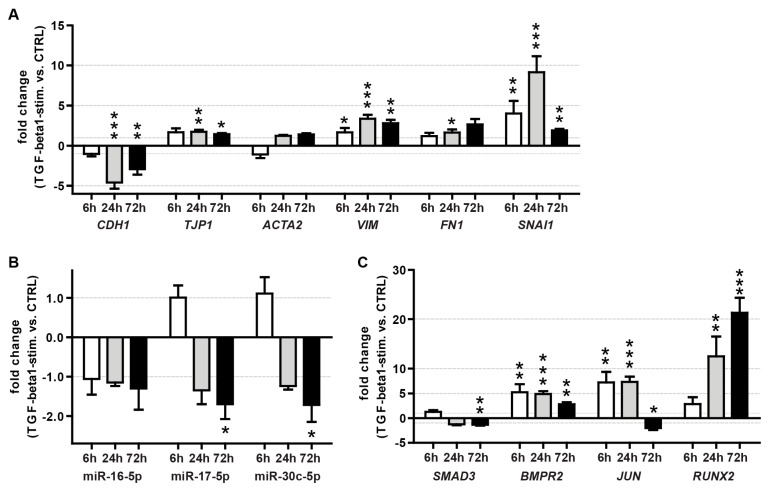
Effect of TGF-β1 treatment on the expression of EMT markers (**A**), miRNAs (**B**) and TGF-β pathway miRNA targets (**C**) in A549 cells. Shown are the mean fold changes (TGF-β1 vs. vehicle control) at 6, 24 and 72 h after stimulation with human recombinant TGF-β1. The arithmetic mean of *RNA18S5* and *HPRT1* mRNA expression served as a normalizer for mRNA quantitation. The small nuclear RNA *RNU6B* served as a reference gene for miRNA quantitation. The results were derived from three independent experiments, with each time point measured in triplicate. T-bars indicate maximum fold changes based on SEMs for target and reference gene expression. Unpaired *t*-test, vs. control treatment: *: *p* < 0.05, **: *p* < 0.01, ***: *p* < 0.001.

Initially, we confirmed TGF-β pathway activation by showing down-regulation of E-Cadherin (*CDH1*) mRNA and up-regulation of the EMT markers vimentin (*VIM*), fibronectin (*FN1*) and snail family zinc finger 1 (*SNAI1*) in A549 cells upon TGF- β1 stimulation (Figure 7A).

To focus on miRNAs with the highest potential relevance for TGF-β regulation, we selected miRNAs with high expression levels (above 20-fold of the median of all expressed miRNAs) (Figure 4) and with ≥3 targets in the TGF-β pathway (Figure 6, Table 4). This resulted in a selection of four miRNAs: miR-16-5p, -17-5p, -24-3p and -30c-5p.

MiRs 17-5p and 30c-5p showed a significant reduction at 72 h (~1.8-fold) after TGF-β1 stimulation compared to the vehicle control, while miR-16-5p and miR-24-3p remained unchanged (Figure 7B).

#### 3.6.2. Effect of TGF-β1 Treatment on Target mRNA Expression in A549 Cells

Due to these results, we speculated that the putative miRNA-based repression of the TGF-β pathway under healthy conditions is released upon stimulation with TGF-β. Moreover, this mechanism, which sustains the homeostasis of TGF-β signaling, might be conserved in humans. Therefore, we also investigated the expression patterns of ATII miRNA targets within the canonical TGF-β pathway upon stimulation with TGF-β1 in A549 cells. While the expression of *BCL2*, *MAP2K4*, *TGFBR2* (data not shown) and *SMAD3* mRNAs was not, or was only mildly, affected by TGF-β1 stimulation. The expression of *BMPR2* (~5.5-fold, ~5-fold and ~2.5-fold for 6 h, 24 h and 72 h treatment, respectively), *JUN* (~15-fold 6 h and 24 h treatment) and *RUNX2* (~12-fold and ~22-fold for 24 h and 72 h treatment, respectively) mRNAs increased drastically within the investigated time frame (Figure 7C). This could indicate that de-repression by down-modulation of miRNAs might, at least in part, play a role in the activation of the TGF-β signaling pathway.

## 4. Discussion

Aberrant miRNA expression has been implicated in the pathogenesis of various ATII-associated diseases [15,16,17,18]. Nonetheless, until now, miRNA expression in healthy controls and respiratory diseases has been mainly studied in cell lines and whole lung samples. Few studies have analysed the expression of various miRNAs in primary ATII cells [19,79,80,81]; however, a complete miRNA expression profile of primary ATII cells has gone unaccounted for. We therefore aimed to provide intact ATII cells for miRNA profiling and to analyse the expression of miRNAs in primary “untouched by antibodies” ATII cells from healthy C57BL/6 mice (commonly used as a model animal for ATII-relevant diseases). We termed the cells “intact ATII cells” because these cells are “untouched by antibodies”; they were isolated by taking advantage of the autofluorescence of this cell type and by staining of the surface markers CD45 of leukocytes and CD31 of endothelial cells. The aim was to establish a preparation method that will provide intact cells for prospective miRNA profiling. Therefore, the isolated cells had to have three main properties: (1) “untouched by antibodies”, (2) high viability and (3) high purity. Thereafter, we aimed to study the regulated pathways of the target mRNAs which could provide an insight into the role of miRNAs in healthy ATII cells.

Studies utilising freshly isolated primary ATII cells are necessary to understand molecular pathways regulating diverse functions of this cell type. However, this research remains highly elusive since the isolation of highly pure, viable and proliferative ATII cells for functional studies is fraught with challenges [82,83,84,85]. First, ATII cells in vitro undergo phenotypic change to resemble ATI cells [61,86,87]. Second, no cell line exists to complement these studies and represent the broad extent of known ATII properties. Therefore, an efficient method for ATII cell isolation is required. Many different isolation methods for ATII cells from mice have been described, including magnetic bead separation [58,88], panning [57,82] and cell-sorting [67,89,90]. However, it is always challenging to isolate highly pure ATII cells. First, extracellular ATII-specific markers for mice are rare and, second, positive selection of ATII cells using ATII-specific markers (CD74 and EpCAM^high^/T1α^neg^) [64,91] could affect certain cellular pathways and therefore change the activation status of purified cells [86,87,92]. It is assumed that EpCAM is involved in diverse intracellular processes, such as cell signaling, migration, differentiation and proliferation [92]. Monoclonal antibodies to EpCAM were described to induce antibody-dependent cellular cytotoxicity in colorectal cancer therapy [86]. The antibody to CD74, which was recently documented as an ATII-specific marker [64], stimulated the cleavage of the CD74 cystolic fragment, inducing NF-ƙB activation [87]. Therefore, a positive selection of ATII cells carries the risk of activating cellular pathways.

We therefore developed a new method for the isolation of ATII cells based on the autofluorescence characteristics of cell populations, thus allowing the isolation of “untouched by antibodies” ATII cells with high viability and purity. It was reported that ATII cells characterized as CD45^neg^/CD31^neg^/Sca-1^neg^/CCSP^neg^ showed high autofluorescence [62]. The presence of a CD45^pos.^ population in our preparations with a slightly higher autofluorescence than that of ATII cells might have indicated macrophages [63]. Autofluorescence arises from endogenous fluorophores which are present in cells and extracellular matrix [89,90]. However, it is not very clear which endogenous fluorophore causes ATII cell autofluorescence. High metabolic activity due to surfactant production and consequently the presence of large amount of metabolic enzymes, such as NAD(P)H and flavins, might contribute to ATII cell autofluorescence [93]. Moreover, porphyrins, present in hemoglobin that has been found in primary ATII cells, exhibit natural autofluorescence [94,95]. However, no study to date has used autofluorescence for the isolation of ATII cells. We used autofluorescence and staining of surface markers of the other cell types to isolate “untouched by antibodies” highly pure sATII cells by FACS. The population of isolated and FACS-sorted ATII cells showed high purity in terms of CD45- and CD31-negativity and CD74-positivity. The few contaminating cells were within the CD45/CD31^neg^ CD74^neg.^ populations. The absence of CD31- and CD45-expressing cells and the expression of epithelial marker proteins (i.e., cytokeratin and E-cadherin) on nearly all sorted cells, as demonstrated by immunofluorescence, confirmed the ATII phenotype of the sorted cells. Expression of CCSP in a few sorted cells might have been due to the presence of Club cells, which have high autofluorescence and co-express proSP-C and CCSP after enzymatic dissociation and sorting [58,96]. Moreover, bronchioalveolar stem cells (BASCs), which develop into bronchiolar and alveolar epithelial cells, also co-express CCSP and proSP-C [19,96]. Nonetheless, they are unlikely to have contaminated the sorted population, as they exhibit low autofluorescence [96]. Another possibility is the presence of ATII progenitor cells that express CCSP and are highly autofluorescent due to their high metabolic activity [97,98]. The existence of endothelial cells post-pATII cell isolation protocol was most likely due to the fact that the “panning” protocol does not use antibodies to deplete endothelial cells.

The purity of the isolated sATII and pATII cells was also confirmed by the high expression and abundance of ATII epithelial and phenotypic markers. Isolated sATII and pATII cells expressed moderate levels of Aqp5, which was in accordance with other studies, showing that murine ATII cells express AQP5, unlike human and rat lung cells, where AQP5 is exclusively ATI-specific [99,100,101,102,103].

In order to dissect the functional role of miRNAs in ATII cells under normal, physiological conditions, miRNA profiling was performed. We used a cut set of miRNAs expressed at similar levels in ATII cells isolated by two different methods. This approach was used in order to identify miRNAs that are common to all ATII cells and not only restricted to distinct ATII cell subsets that are enriched by one of the isolation methods. Furthermore, this method reduces the activation of pathways which could be triggered during the isolation process and thus minimizes the changes in miRNA expression. Enrichment of target mRNAs with binding sites for cut set of ATII miRNAs in distinct pathways argues for biological relevance of a given pathway in these cells. This way, miRNAs can serve as a tool to detect or prioritize important pathways that might be overlooked in primarily mRNA-based identification strategies.

A cut set of 111 ATII miRNAs was used for pathway enrichment analysis in order to identify miRNA-regulated pathways involved in ATII cell homeostasis. Of 145 classified pathways with statistically significant target enrichment, only two pathways regulate metabolic processes. This could indicate that under normal physiological conditions, miRNAs in ATII cells may not play an important role in metabolic pathway regulation. However, since our approach was limited to miRNA–target interactions that were experimentally observed to date, it is possible that more miRNA targets and relevant pathways may be identified in ATII cells in the future.

The role of miRNAs was further confirmed by pathway analysis, which revealed that top network functions of the ATII miRNA target gene set were associated with pathways related to “cancer” and to “fibrosis and/or EMT”. These findings are in agreement with those of a study by Fujino and colleagues, who showed that ATII cells isolated from human biopsy samples expressed genes enriched for positive regulation of cell differentiation and lung development [91]. Further, these results are in accordance with a recent study by Zacharias and colleagues [104]. Specific molecular pathways that have been already associated with these functions in ATII cells include TGF-β, Wnt/β-Catenin and growth factor signaling (e.g., EGF, HGF, KGF) [59,93,105,106]. In line with these studies, we have found that TGF-β and EGF are among the top five upstream regulators. Among miRNAs expressed above the median level, 16 miRNAs have their targets in the canonical TGF-β pathway, such that two of these miRNAs are within the top five upstream regulators. In this context, TGF-β is a potent EMT inducer that functions in cellular proliferation and differentiation, as well as in apoptosis, and therefore plays a crucial role in the regulation of epithelial homeostasis [107,108,109]. Similarly, the EGF protein family promotes EMT by stimulation of alveolar epithelial cell proliferation and migration [107,110]. Therefore, it has been suggested that there is cross-talk between these growth factors within the TGF-β pathway; however, the exact mechanism is unknown [105,106,111,112].

Several reports have shown that miRNAs are able to regulate these pathways. We found that miR-30a-3p/5p, miR-30c-5p and miR-30e-3p/5p were amongst those with the highest expression in ATII cells. Along this line, miR-30c-5p was among the top upstream regulators with three targets within the TGF-β pathway, while miRs 30a/e-3p were the most abundant. Down-regulation of miR-30 was observed in lung samples from IPF and NSCLC patients [13,79]. Transfection of hepatocyte cell line AML12 with these miRNAs resulted in decreased TGF-β1-induced EMT, while TGF-β1 treatment resulted in down-regulation of these miRNAs [113]. Moreover, Zhou and colleagues further showed that miR-30a down-regulates TGF-β1-induced EMT and peritoneal dialysis-related peritoneal fibrosis through down-regulation of snai1 [114]. Therefore, the high expression of three miR-30 family members in the present study could suggest that this family plays a crucial role in suppressing EMT in ATII cells under normal physiological conditions. Four members of the miR17~92 cluster (miR-19a, -17, -20a and 18a) revealed high to moderate expression in ATII cells in our study, with miR-17-5p having three targets within the canonical TGF-beta signaling pathway. The activation of this cluster in neuroblastoma cells was reported to regulate TGF-β signaling components [115]. In addition, it was reported that this cluster regulates cell proliferation and collagen synthesis by targeting the TGF-β pathway [116]. Further, the current study shows that the expression of miR-16-5p is among the top upstream regulators, with four targets within the TGF-β pathway, thus suggesting a crucial role for this miRNA in ATII homeostasis. It was shown that overexpression of miR-16 inhibited EMT-mediated factors Snail and Twist in vitro in a prostate cancer cell line [117]. It was reported that p53, a tumor suppressor, induces miR-16, whereas the down-regulation of p53 leads to EMT-related stem cell phenotypes [118,119], suggesting that miRNAs are regulators of the p53-controlled epithelial phenotype in ATII cells under normal physiological conditions. Hence, miRNAs are important in protection from fibrosis and cancer progression and in the maintenance of the ATII cell phenotype.

The miR-200 family member, miR-429, was also strongly expressed in our ATII cell preparation. ATII cells play an important role in the pathogenesis of IPF due to loss of their regenerative capacity [120,121]. Moreover, ATII cells isolated from IPF patients demonstrate impairment in their transdifferentiation into ATI cells [122], which triggers dysfunction in epithelial–mesenchymal transition (EMT) in the alveolar epithelium and leads to fibrosis [83,123]. miR-200 family members were reported to control these pathways, such that they were shown to be down-regulated in the lungs of IPF patients as well as in mice with experimental pulmonary fibrosis [15]. Interestingly, it was described that miR-200 family can restore normal regenerative function in exhausted senescent IPF pneumocytes by induction of transdifferentiation of primary human IPF ATII cells into ATI cells [81]. Moimas et al. [81] have demonstrated that upon transfection of IPF ATII cells with synthetic mimics of the entire miR-200 family, i.e., with miR-200b-3p and miR-200c-3p, they were able to restore the capability of exhausted senescent IPF ATII cells to transdifferentiate into ATI cells. It was shown that miR-429 reversed EMT in metastatic ovarian cancer cells [113] and was down-regulated in TGF-β1 treated MDCK cells [124]. The miR-200 family is a well-known inhibitor of TGF-β-induced EMT, and it is highly expressed in almost all epithelial cell types, except cells of mesenchymal origin [125,126,127]. Studies have shown that low expression of miR-200 family members is associated with poor prognosis in cancers, such as ovarian, gastric and thyroid cancers and many more [128,129,130,131]. Moreover, Tellez and colleagues further reported that miR-200 family members were repressed in immortalized human bronchial epithelial cells during EMT induced by tobacco carcinogens [132]. However, the overexpression of miR-200 family members in a lung adenocarcinoma mouse model restricted the cancer cells to an epithelial phenotype and stopped metastases [133]. In line with this, various functional studies showed that the down-regulation of miR-200 induced EMT, whereas its overexpression provoked mesenchymal-to-epithelial transition (MET) and inhibited cancer cell motility by repression of ZEB1 and ZEB2 [127,134,135,136]. Thus, both transcriptional factors could be involved in the TGF-β- pathway via a negative loop with miR-200. Therefore, the expression of miR-30- and miR-200 family members shows that ATII miRNAs play an important role in maintaining epithelial homeostasis. The finding that miRNAs targeting TGF-β signaling components are down-regulated by TGF-β is in accordance with our findings for down-modulation of miR-17-5p and -30c-5p in TGF-β1-treated A549 cells and supports the idea that under physiological conditions this pathway is at least partially controlled by miRNAs.

Recent studies have indicated that downstream molecules of the TGF-β signaling pathway interfere with miRNA expression either by regulating their transcriptional or post-transcriptional processing via interaction with components of the miRNA biogenesis machinery or by modulating epigenetic marks on miRNA promoters [137,138]. Additionally, miRNAs target components of the TGF-β signaling pathway, resulting in a complex network of signaling loops that contribute to the modulation of this pathway (reviewed in [139,140]). Since primary ATII cells gradually lose their phenotypes during in vitro culture, we decided to study the effects of TGF-β stimulation in the human alveolar epithelial cell line A549 that features hallmark characteristics of ATII cells [141].

MiR-16-5p and miR-30c-5p are top upstream regulators of the investigated set of target genes and have four (MAP2K1, MAP2K4, JUN and BCL2) and three (ACVR1, JUN and RUNX2) experimentally observed targets in the TGF-β signaling pathway, respectively. MiR-17-5p has three experimentally observed targets in the TGF-β signaling pathway (TGFBR2, BMPR2 and BCL2) and is a member of the miR-17~92 cluster, which has been associated with inhibition of TGF-β signaling. Most importantly, all of these ATII miRNAs have been associated with inhibition of TGF-β signaling [113,115,142,143,144,145]. Additionally, Corcoran et al. [146] reported that a set of miRNAs expressed in ATII cells is down-regulated in A549 cells upon TGF-β stimulation. These findings are in agreement with our findings regarding the down-regulation of miR-30c and miR-17-5p in A549 cells. Furthermore, it was shown that TGF-β-induced target gene expression is tightly controlled through down-regulation of miRNAs via TGFβ-induced transcription factors, such as AP-1, SMAD3/4 and NF-κB [146]. Of note, we found that TGF-β1 is a predicted upstream regulator for the set of validated mRNA targets interacting with the ATII-expressed miRNAs, of which 19 had experimentally observed targets in the canonical TGF-β pathway of the Ingenuity^®^ database. This finding supports the idea of a complex interaction between TGF-β signaling and miR regulation in ATII cells already under normal conditions, hence underlining the importance of miRNAs for sustaining TGF-β pathway homeostasis. Additionally, Pandit et al. showed the down-regulation of 18 miRNAs—7 of which were also expressed in our ATII cells—in lung tissues of patients with idiopathic pulmonary fibrosis, indicating that disturbance of this mechanism might contribute to disease progression [79]. Finally, homeostatic down-modulation of TGF-β signaling by miRNAs expressed under normal conditions might contribute to the inhibition of ATII to ATI trans-differentiation [147].

In summary, these findings suggest that autofluorescence characteristics of murine lung cells can be exploited to isolate highly pure, untouched ATII cells and that miRNAs expressed in ATII cells contribute to cellular homeostasis by the modulation of proliferation and cell-activation pathways. Based on our data for miRNA expression in ATII cells, under normal conditions, and enrichment of miRNA targets in the TGF-β pathway, we hypothesize that miRNAs might represent valuable tools for the early detection of pathological conditions, such as fibrotic lung diseases and lung cancer.

## Figures and Tables

**Table 1:** Antibodies used in this study.

**Antibodies for Flow Cytometry and Cell Sorting (ITC: Isotype Control):**					
**Antigen**	**Host**	**Isotype**	**Fluorochrome**	**Clone**	**Comp** **any**
CD31	Rat	IgG2a, k	APC	MEC 13.3	BD Pharmingen
ITC for CD31	Rat	IgG2a, k	APC	R35-95	BD Pharmingen
CD31	Rat	IgG2a, k	PE	390	BioLegend
ITC for CD31	Rat	IgG2a, k	PE	RTK2758	BioLegend
CD45	Rat	IgG2b, k	APC	30-F11	BD Pharmingen
ITC for CD45	Rat	IgG2b, k	APC	A95-1	BD Pharmingen
CD74	Rat	IgG2b, k	FITC	In-1	BD Pharmingen
ITC for CD74	Rat	IgG2b, k	FITC	A95-1	BD Pharmingen
**Primary Antibodies for Immunofluorescence Staining:**					
**Antigen**	**Host**	**Isotype**	**Clone**	**Company**	
Pan-cytokeratin	Goat	IgG1	C-11	Abcam	
E-Cadherin	Mouse	IgG2a, k	36/E-Cadherin	BD Pharmingen	
Alpha-SMA	Mouse	IgG2a	1A4	Sigma	
CD31	Rabbit	IgG	Polyclonal	Abcam	
Pro-SPC	Rabbit	IgG	Polyclonal	Chemicon/Millipore	
CCSP	Rabbit	IgG	Polyclonal	Upstate/Millipore	
CD45	Rat	IgG2b, k	30-F11	BD Pharmingen	
**Secondary Antibodies for Immunofluorescence Staining:**					
**Antigen**	**Host**	**Isotype**	**Fluorochrome**	**Company**	
Rabbit-IgG (H+L)	Goat	IgG	Alexa Fluor 555	Invitrogen	
Mouse-IgG (H+L)	Goat	IgG	Alexa Fluor 555	Inivtrogen	
Rat-IgG (H+L)	Goat	IgG	Alexa Fluor 555	Inivtrogen	
Goat-IgG (H+L)	Donkey	IgG	Alexa Fluor	Inivtrogen	

* The CD31-PE antibody used recognizes a different epitope of CD31 than the CD31-APC antibody used for sorting.

**Table 2:** Primers used for RT-qPCR.

Gene Symbol	Species	NCBI GenBank Accession	Primers (5′->3′)	Product Size (bp)
Acta2	Mmu	NM_007392	Fwd: GCTGGTGATGATGCTCCCA Rev: GCCCATTCCAACCATTACTCC	81
Aqp5	Mmu	NM_009701	Fwd: CCTTATCCATTGGCTTGTCG Rev: CTGAACCGATTCATGACCAC	115
Cd74	Mmu	NM_001042605	Fwd: GATGGCTACTCCCTTGCTGA Rev: TGGGTCATGTTGCCGTACT	93
Cdh1	Mmu	NM_009864	Fwd: CCATCCTCGGAATCCTTGG Rev: TTTGACCACCGTTCTCCTCC	89
Hprt	Mmu	NM_013556	Fwd: CCTAAGATGAGCGCAAGTTGAA Rev: CCACAGGACTAGAACACCTGCTAA	86
Pecam1	Mmu	NM_008816	Fwd: ATCGGCAAAGTGGTCAAGAG Rev: GGCATGTCCTTTTATGATCTCAG	111
Ptprc	Mmu	NM_001111316	Fwd: GTCCCTACTTGCCTATGTCAATG Rev: CCGGGAGGTTTTCATTCC	115
Sftpa1	Mmu	NM_023134	Fwd: GGAGAGCCTGGAGAAAGGGGGC Rev: ATCCTTGCAAGCTGAGGACTCCC	124
Sftpc	Mmu	NM_011359	Fwd: AGCAAAGAGGTCCTGATGGA Rev: GAGCAGAGCCCCTACAATCA	153
Tjp1	Mmu	NM_009386	Fwd: ACGAGATGCTGGGACTGACC Rev: AACCGCATTTGGCGTTACAT	112
*ACTA2*	HSA	NM_001141945	Fwd: GGCTCTGGGCTCTGTAAGG Rev: TTTGCTCTGTGCTTCGTCAC	147
*BCL2*	HSA	NM_000633	Fwd: CTGAGTACCTGAACCGGCA Rev: GAGAAATCAAACAGAGGCCG	106
*BMPR2*	HSA	NM_001204	Fwd: TGCCCTCCTGATTCTTGG Rev: CATAGCCGTTCTTGATTCTGC	130
*CDH1*	HSA	NM_004360	Fwd: ATACACTCTCTTCTCTCACGCTGTGT Rev: CATTCTGATCGGTTACCGTGATC	89
*FN1*	HSA	NM_212482	Fwd: CCGACCAGAAGTTTGGGTTCT Rev: CAATGCGGTACATGACCCCT	81
*HPRT1*	HSA	NM_000194	Fwd: TTGTTGTAGGATATGCCCTTGAC Rev: TCTCATCTTAGGCTTTGTATTTTGC	105
*JUN*	HSA	NM_002228	Fwd: CAGAGAGACAGACTTGAGAACTTGAC Rev: GACGCAACCCAGTCCAAC	100
*MAP2K4*	HSA	NM_003010	Fwd: GGCCAAAGTATAAAGAGCTTCTGA Rev: CAGCGATATCAATCGACATACAT	145
*RNA18S5*	HSA	NR_003286	Fwd: GCAATTATTCCCCATGAACG Rev: AGGGCCTCACTAAACCATCC	125
*RUNX2*	HSA	NM_001024630	Fwd: TAGATGGACCTCGGGAACC Rev: GAGGCGGTCAGAGAACAAAC	77
*SMAD3*	HSA	NM_005902	Fwd: GTCAAGAGCCTGGTCAAGAAAC Rev: GATGGGACACCTGCAACC	136
*SNAI1*	HSA	NM_005985	Fwd: CTTCTCTAGGCCCTGGCTG Rev: AGGTTGGAGCGGTCAGC	105
*TGFBR2*	HSA	NM_001024847	Fwd: TCTGTGGATGACCTGGCTAAC Rev: TCATTTCCCAGAGCACCAG	148
*TJP1*	HSA	NM_003257	Fwd: GAGGAAACAGCTATATGGGAACAAC Rev: TGACGTTTCCCCACTCTGAAA	120
*VIM*	HSA	NM_003380	Fwd: AGATGGCCCTTGACATTGAG Rev: TGAGTGGGTATCAACCAGAGG	146

**Table 3:** Categories of pathways with significant ATII miR–target enrichment.

Pathway Category	Pathways per Category	Examples of Pathways within Category
Cancer	30	Small and non-small cell lung cancer, p53
Cellular growth, proliferation and development	28	PI3K/Akt, ILK, TGF-β, Integrin, FAK, mTOR
Cytokine signaling	27	Chemokine, IL-6, IL-8, IL-9, IL-10, IL-15, IL-17, IL-22, TNFR1
Cellular immune response	22	CXCR4, HMGB1, NF-κB, dendritic cell maturation
Growth factor signaling	21	IGF-1, EGF, GM-CSF, VEGF, FGF, PDGF
Apoptosis signaling	16	PTEN, death receptor, 14-3-3, JAK/Stat, tight junction signaling
Cell cycle regulation	13	G1/S checkpoint regulation, G2/M DNA damage checkpoint regulation
Intracellular and second messenger	13	Glucocorticoid receptor, ERK/MAPK, Rac, Rho, Gα12/13, PAK
Neurotransmitters and other nervous system signaling	13	Neuregulin, ErbB, Ephrin receptor, axonal guidance
Organismal growth and development	13	Stem cell pluripotency, HGF, BMP, Wnt/β-catenin
Disease-specific pathways	9	Hepatic fibrosis, rheumatoid arthritis, Huntington’s disease
Cardiovascular signaling	7	Cardiac hypertrophy, atherosclerosis, thrombin signaling
Cellular stress and injury	6	HMGB1, HIF1α, p70S6K
Humoral immune response	5	CD40, IL-4, B cell receptor signaling
Nuclear receptor signaling	5	PPARα/RXRα activation, PPAR, RAR activation, VDR/RXR activation
Pathogen-influenced	3	LPS-stimulated MAPK signaling
Transcriptional regulation	2	Role of NANOG and Oct4 in mammalian embryonic stem cell pluripotency
Xenobiotic metabolism	1	Aryl hydrocarbon receptor signaling
Metabolism of cofactors and vitamins	1	Nicotinate and nicotinamide metabolism
Metabolism of complex lipids	1	Inositol phosphate metabolism

**Table 4:** Mapping of ATII-expressed miRNAs to TGF-β pathway signaling components.

miRNA	miRBase MIMAT ID	Number of Targets	Pubmed ID for Exp. Obs. Interaction	mRNA Target	Transduction Level, Molecular Type
Mmu-miR-22-3p	0000531	1	19011694	*Bmp7*	Extracellular ligand, growth factor
Mmu-miR-29a-3p	0000535	2	19342382	*Tgfb3*	
Mmu-miR-30c-5p	0000514	3	18258830	*Acvr1*	Plasma membrane receptor, kinase
Mmu-miR-24-3p	0000219	6	17906079	*Acvr1b*	
Mmu-miR-210-3p	0000658	1	19520079	*Acvr1b*	
Mmu-miR-29a-3p	0000535	2	19342382	*Acvr2a*	
Mmu-miR-125a-5p	0000135	1	19738052	*Bmpr1b*	
Mmu-miR-19a-3p	0000651	1	19390056	*Bmpr2*	
Mmu-miR-25-3p	0000652	2	19390056	*Bmpr2*	
Mmu-miR-17-5p	0000649	3	19390056	*Bmpr2*	
Mmu-miR-17-5p	0000649	3	20709030	*Tgfbr2*	
Mmu-miR-18a-3p	0004626	1	19372139	*Kras*	Cytoplasmatic signaling, enzyme
Mmu-miR-181a-5p	0000210	2	20080834	*Kras*	
Mmu-miR-16-5p	0000527	4	20065103	*Map2k1*	Cytoplasmatic signaling, kinase
Mmu-miR-16-5p	0000527	4	19861690	*Map2k4*	
Mmu-miR-24-3p	0000219	6	19861690	*Map2k4*	
Mmu-miR-25-3p	0000652	2	19861690	*Map2k4*	
Mmu-miR-24-3p	0000219	6	19502786	*Mapk14*	
Mmu-miR-7a-5p	0000677	2	19072608	*Raf1*	
Mmu-miR-199a-3p	0000230	1	19251704	*Smad1*	Transcription factor
Mmu-miR-23b-3p	0000125	3	19582816	*Smad3*	
Mmu-miR-24-3p	0000219	6	19582816	*Smad3*	
Mmu-miR-27a-3p	0000537	3	19582816	*Smad3*	
Mmu-miR-140-5p	0000151	1	20071455	*Smad3*	
Mmu-miR-23b-3p	0000125	3	19582816	*Smad4*	
Mmu-miR-24-3p	0000219	6	19582816	*Smad4*	
Mmu-miR-27a-3p	0000537	3	19582816	*Smad4*	
Mmu-miR-23b-3p	0000125	3	19582816	*Smad5*	
Mmu-miR-24-3p	0000219	6	19582816	*Smad5*	
Mmu-miR-27a-3p	0000537	3	19582816	*Smad5*	
Mmu-miR-7a-5p	0000677	2	17028171	*Fos*	
Mmu-miR-222-3p	0000670	1	20299489	*Fos*	
Mmu-miR-16-5p	0000527	4	18362358	*Jun*	
Mmu-miR-30c-5p	0000514	3	18668040	*Jun*	
Mmu-miR-30c-5p	0000514	3	21628588	*Runx2*	
Mmu-miR-218-5p	0000663	1	21628588	*Runx2*	
Mmu-miR-16-5p	0000527	4	18449891	*Bcl2*	Transcription factor target, transporter
Mmu-miR-17-5p	0000649	3	19666108	*Bcl2*	
Mmu-miR-181a-5p	0000210	2	20204284	*Bcl2*	

## Supplementary Materials

The following are available online at https://www.mdpi.com/article/10.3390/genes13081420/s1, Table S1: Upstream regulators. Table S2: ATII miR expression profile.
